# The scanning CONfoCal Ophthalmoscopy foR DIAbetic eye screening (CONCORDIA) study paper 2

**DOI:** 10.1038/s41433-024-03361-1

**Published:** 2024-10-11

**Authors:** Peter H. Scanlon, Marta Gruszka-Goh, Ushna Javed, Anthony Vukic, Julie Hapeshi, Steve Chave, Paul Galsworthy, Scott Vallance, Stephen J. Aldington

**Affiliations:** 1 Gloucestershire Retinal Research Group (GRRG), Cheltenham General Hospital, Cheltenham, UK; 2 Nuffield Department of Clinical Neuroscience, University of Oxford, Oxford, UK; 3 University of Gloucestershire, Cheltenham, UK; 4 The Royal College of Ophthalmologists’ National Ophthalmology Audit, London, UK

**Keywords:** Public health, Medical imaging

## Abstract

**Purpose:**

To determine if the Eidon white light 60-degree field Scanning Confocal Ophthalmoscope (SCO) camera was safe to use with staged mydriasis in a Diabetic Eye Screening Programme (DESP).

**Methods:**

The trial participants were recruited from people with diabetes attending appointments in DESP or Virtual Eye clinics for post-Covid delayed hospital appointments. Using staged mydriasis, the SCO images were taken before the pupils were dilated and compared to two-field 45 degrees mydriatic digital photography (the reference standard). Mydriatic SCO images were only compared to the reference standard if the non-mydriatic SCO images were unassessable.

**Results:**

1050 patients were recruited, 35 individuals were withdrawn, the majority (18) due to an imaging protocol deviation leaving 1015 individuals (2029 eyes). Using staged mydriasis, the sensitivity and specificity for any retinopathy was 97.5% (95% CI: 96.4–98.4%) and 82.3% (95% CI: 79.6–84.7%) respectively. The sensitivity and specificity for referable retinopathy was 92.7% (95% CI: 89.9–94.9%) and 85.4% (95% CI: 83.6–87.2%) respectively. The total number of eyes that were unassessable with the Eidon without mydriasis was 85/2029 (4.2%), and after mydriasis was 34/2029 (1.7%) and, with the reference standard, 34/2029 (1.7% - not always the same images) were unassessable.

**Conclusions:**

This study provides promising early results of the performance of the Eidon camera using staged mydriasis in a DESP which needs further evidence from a non-Caucasian population and from cost-effectiveness analyses.

## Introduction

The CONCORDIA (Scanning **CON**fo**C**al **O**phthalmoscopy fo**R DIA**betic eye screening) study is a programme of research to look at the accuracy of Scanning Confocal Ophthalmoscope (SCO) and Broad Line Fundus Imaging camera devices in detection of any eye disease caused by diabetes. The over-arching objective of this work is to determine if these cameras can be used in the non-mydriatic mode, and to determine if they are cost-effective to use in the NHS Diabetic Eye Screening Programme (DESP).

The subject of this paper, is a clinical trial to determine the sensitivity and specificity of detection of any retinopathy and of referable retinopathy, using the Eidon (Centervue, Padova, Italy) SCO camera, that has a 60 degree field, against the reference standard of two-field digital imaging used by the NHS diabetic eye screening programme in the area photographed by the two 45-degree fields.

In 2019, Sarao et al. [1] compared the Eidon white LED confocal camera and a traditional flash fundus camera (TRCNW8, Topcon Corporation, Tokyo, Japan) that were used to capture fundus images. Colour images were evaluated with respect to chromaticity. Analysis was performed according to the image colour signature. The colour signature of an image was defined as the distribution of its pixels in the RGB chromaticity space. The conclusion of the study was that the Eidon provides more-balanced colour images, with a wider richness of colour content, compared to a conventional flash fundus camera.

In 2020, Olvera-Barrios et al. [2] compared 1257 people with diabetes attending the NHS Diabetic Eye Screening Programme comparing Diabetic Retinopathy (DR) grades from two 45-degree field mydriatic digital imaging and the Eidon single mydriatic 60-degree image. Agreement after consensus with kappa statistic was 0.89 (quadratic weights (95% CI 0.87–0.92)) for NDESP severity grade, 0.88 (quadratic weights (95% CI 0.82–0.94)) for referable disease and 0.92 (linear weights (95% CI 0.88–0.95)) for maculopathy.

In 2022, Ashraf et al. [3] published results of a study of one hundred and ten eyes of 55 patients with diabetes mellitus designed to evaluate agreement of non-mydriatic 4 fields with the Eidon camera and mydriatic photos from the Optos camera for identification of diabetic retinopathy (DR) severity. Before grading, a standardized Early Treatment Diabetic Retinopathy Study [4] 7-field image mask was applied to all Optos retinal images. Sensitivity and specificity compared with ETDRS field grading for any DR were 0.96 and 0.75, for moderate NPDR or worse were 0.96 and 0.97, and for severe NPDR or worse were 0.91 and 1.00, respectively.

## Methods

**Fig. 1:**
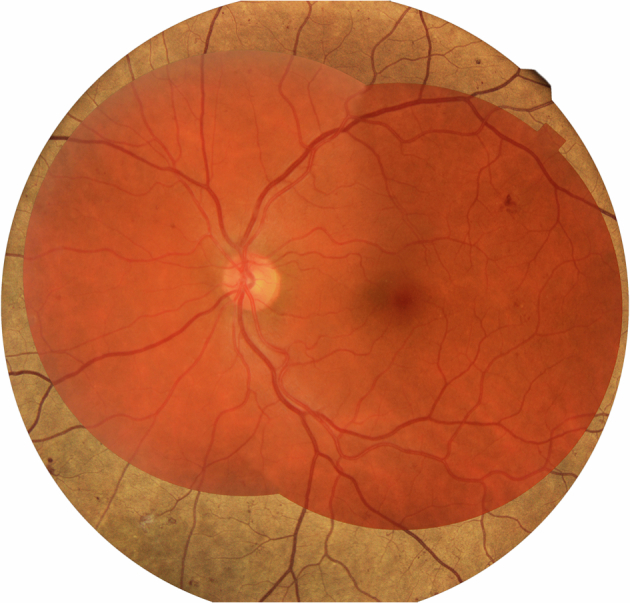
Eidon 60-degree image superimposed over the area captured by two 45-degree fields.

We proposed a clinical trial to determine the sensitivity and specificity of the Eidon SCO camera using a staged mydriatic approach in a screening cohort setting to detect retinopathy lesions in the central area covered by the standard two 45-degree photographs that is currently used in the NHS Diabetic Eye Screening Programme. The staged mydriatic approach was chosen because there would be considerable advantages in using the SCO devices if the majority of patients screened did not need their pupils dilated to take an assessable image and their use was found to be cost-effective. The Eidon camera captures one field 60-degree image per eye with a white light LED source that covers slightly more than the area covered by the two 45-degree digital photographs taken by the conventional cameras used in the English NHS DESP (Fig. 1).

The participants for the AIDED and CONCORDIA studies were recruited during routine digital screening and digital surveillance clinics run by the Gloucestershire DESP following all the protocols of the English NHS DESP. Study participants were also recruited from virtual eye clinics which had been set up to cope with the backlog of patients whose hospital ophthalmology follow up appointments were delayed because of the Covid-19 epidemic. Because these patients had previously been referred into the hospital, they had a higher proportion of referable retinopathy than those in the routine digital screening clinics. Fundus imaging protocols within the virtual eye clinic were synonymous to DESP standards.

Reference standard photographs were taken using non-mydriatic digital cameras used by the Gloucestershire DESP which have been approved for use in the NHS DESP. The cameras used in the Gloucestershire DESP are the Canon CR2, Kowa AF, Topcon Triton and Topcon 2000.

When image quality was poor or pupil size was less than 3 mm, images were taken before and after dilation with the SCO device. The screeners in the English screening programme are not currently trained to assess whether an image is assessable at the point of capture. Eidon marketing materials suggest 2.5 mm is their minimum capture range, and it was decided to take a mydriatic image with the SCO cameras if the image was very obviously unassessable or if the pupil size was less than 3 mm. The Eidon camera has a measurement function for pupil size. If the non-mydriatic images were assessable, these images were used in comparison to the 2-field mydriatic digital images otherwise the mydriatic image was utilised.

All images were graded by two graders with arbitration for differences of opinion by a third grader. The graders determined the retinopathy (R), maculopathy (M) and presence of photocoagulation scars (P) levels and also whether the images were assessable or not. Where images had been taken with and without mydriasis (e.g., in a patient with a pupil size less than 3 mm) and the non-mydriatic image was considered assessable by the graders, this was the image used for the comparisons of microaneurysm counts and for the sensitivity and specificity levels of any and referable diabetic retinopathy (DR).

All graders were either senior graders in the Gloucestershire DESP or graders in the English NHS DESP who are members of the Grading College, both of whom score highly in the monthly quality assurance Test and Training (TaT) sets that are taken by graders in the English NHS DESP. The Grading College members are trusted to provide the guide grade for the TaT sets because of high scores of their grading in the previous 12 months.

The senior graders in the Gloucestershire Retinal Research Group (GRRG) have a track record of grading for a number of studies including a Health Technology Assessment report [5] on optimisation of the screening interval in diabetic retinopathy screening and the CLEOPATRA study [6].

The graders used both colour and red free tools to assess the images. All images were converted to red free as part of the grading process.

The Grading Form, which is based on the grading form used by the NHS Diabetic Eye Screening Programme in England is displayed in Supplementary Table 1.

Supplementary Tables 2 and 3 compare the English NHS DESP grading form with the Early Treatment Diabetic Retinopathy Study (ETDRS) and International Classifications for Diabetic Retinopathy and Maculopathy respectively.

The graders were not aware of the source of the images i.e. which type of clinic the images came from. Images were allocated to individual graders in batches depending on grader availability with certain individuals allocated to Eidon images and others to digital images. No grader graded images from both cameras on the same patient.

To compare the same areas of the eyes, SCO images were graded with a two 45-degree field overlay and all images graded using the NHS DESP grading criteria. In addition, microaneurysm counts were undertaken in the area of the 45-degree central macular field overlay. For the purposes of the analysis, the microaneurysm counts within 1 DD of the central fovea were used for comparison. They were recorded as 0, 1, 2, 3, 4, 5+ and double graded and arbitrated. Ethics approval for the CONCORDIA study was granted by the Health Research Authority with IRAS project ID: 297725. REC reference: 21/SW/0064.

The clinical trial reference number was ISRCTN16254044.

The sample size was calculated using sample size formulae for calculating adequate sensitivity/specificity from Hajian-Tilaki [7].

Characteristics of the population, by baseline DR severity and analysis cohorts, were summarized using descriptive statistics.

Sensitivity and specificity comparing SCO device with the reference standard were calculated for any and referable levels of DR. The unassessable pictures from the reference standard were excluded from the sensitivity, specificity, positive and negative predictive value analysis. All statistics were calculated with exact binomial confidence intervals.

## Results

1050 patients were recruited as trial participants from clinics of the Gloucestershire Diabetic Eye Screening Programme and Virtual Eye clinics for patients whose hospital eye service appointments were delayed due to Covid-19. Informed consent was obtained from all participating subjects. 35 individuals were withdrawn for the following reasons:

- Imaging protocol deviation /camera error - 18.
- Patient withdrew consent and left before the imaging was complete – 9.
- IT network error – 1.
- Recruitment/administrative error e.g., patient study ID not allocated – 7.

The final sample consisted of 1015 individuals (2029 eyes).

The CONCORDIA Eidon trial cohort consisted of 1015 patients (2029 eyes). 603 (59.4%) of patients were male and 412 (40.6%) female.

The median age at the time of trial was 64.0 years with IQR (55.0–73.0) years and was two years higher for male patients. The most frequent (29.5%) trial participants were patients aged 65 to 74 years and the least frequent (1.1%) those aged 24 years or younger.

The large majority (93.2%) of patients were of White Caucasian descent and proportions of individuals from another ethnic group ranged between 1.5% and 3.5%, Table 1.

**Table 1:** Baseline characteristics of the trial patients.

	Gender		
	Female	Male	Total
*N*	412 (40.6%)	603 (59.4%)	1015 (100.0%)
Median Age (years)	63.0 (53.0–72.0)	65.0 (55.0–73.0)	64.0 (55.0–73.0)
Age Category (years)			
12–24	5 (1.2%)	6 (1.0%)	11 (1.1%)
25–34	19 (4.6%)	14 (2.3%)	33 (3.3%)
35–44	38 (9.2%)	42 (7.0%)	80 (7.9%)
45–54	48 (11.7%)	81 (13.4%)	129 (12.7%)
55–64	112 (27.2%)	156 (25.9%)	268 (26.4%)
65–74	106 (25.7%)	193 (32.0%)	299 (29.5%)
75–84	67 (16.3%)	99 (16.4%)	166 (16.4%)
85+	17 (4.1%)	12 (2.0%)	29 (2.9%)
Ethnicity			
White Caucasian	384 (93.2%)	562 (93.2%)	946 (93.2%)
Asian or Asian British	12 (2.9%)	24 (4.0%)	36 (3.5%)
Black or Black British	5 (1.2%)	10 (1.7%)	15 (1.5%)
Mixed - Multiple Ethnic Groups	10 (2.4%)	6 (1.0%)	16 (1.6%)
Data Not Available	1 (0.2%)	1 (0.2%)	2 (0.2%)

Out of 2029 images taken using Eidon, 1944 (95.8%) were assessable without mydriasis and 85 (4.2%) (95% CI: 3.4–5.2%) were unassessable according to the grader.

The percentage of assessable eyes after dilation using the Eidon increased to 1995 (98.3%) with 34 (1.7%) (95% CI:1.2–2.3%) were unassessable according to the grader.

Similarly, out of 2029 photos taken post-dilation using 2 × 45 degree field digital photography, 34 (1.7%) (95% CI:1.2–2.3%) were unassessable according to the grader.

No patient under the age of 34 had an unassessable eye in non-mydriatic Eidon imaging and less than 3% under the age of 55. The age group of patients whose eyes were unassessable is shown in Table 2:

**Table 2:** Age group of patients whose eyes were unassessable.

		Eidon pre-dilation		Eidon staged-dilation		2-field mydriatic digital photography	
	Overall number of eyes	Number of eyes	Percentage of eyes	Number of eyes	Percentage of eyes	Number of eyes	Percentage of eyes
Age category (years)							
12–24	22	0	0.0%	0	0.0%	0	0.0%
25–34	66	0	0.0%	0	0.0%	0	0.0%
35–44	160	4	2.5%	3	1.9%	0	0.0%
45–54	258	7	2.7%	6	2.3%	2	0.8%
55–64	535	13	2.4%	6	1.1%	7	1.3%
65–74	598	35	5.9%	9	1.5%	16	2.7%
75–84	332	23	6.9%	9	2.7%	9	2.7%
85+	58	3	5.2%	1	1.7%	0	0.0%
Total	2029	85	4.2%	34	1.7%	34	1.7%

Of the 34 eyes considered unassessable, the staged-mydriatic Eidon and 2 × 45-degree field mydriatic digital photos agreed that 8 eyes were unassessable on both devices and, in 52 eyes they differed in unassessability, leaving 1969 eyes that were assessable on both devices after dilation.

As the reference standard was 2-field mydriatic digital photography, we estimated the sensitivity and specificity of the staged mydriatic approach using the Eidon on 1008 patients (1969 eyes) because these were the eyes considered assessable on both the Eidon with staged mydriasis and the reference standard of 2 ×45-degree mydriatic digital photography. Grading results are shown in Table 3.

**Table 3:** Grading results.

		Reference standard														
		No DR	Non referable DR		Referable DR											Total (%)
Staged mydriasis Eidon		R0M0	R1M0 P0	R1M0 P1	R1M1 P0	R1M1 P1	R2M0 P0	R2M0 P1	R2M1 P0	R2M1 P1	R3M0 P0	R3M0 P1	R3M1 P0	R3M1 P1	R3MU P1^a^	
No DR	R0M0	729	25	0	1	0	0	0	0	0	0	1	0	0	0	756 (38.4%)
Non-Referable DR	R1M0 P0	128	388	0	30	0	1	0	0	0	0	0	0	0	0	547 (27.8%)
	R1M0 P1	4	2	10	0	0	0	0	0	0	0	1	0	0	0	17 (0.9%)
Referable DR	R1M1 P0	18	92	0	204	0	0	0	2	0	0	0	0	0	0	316 (16.1%)
	R1M1 P1	0	1	0	3	3	0	0	0	0	0	0	0	0	0	7 (0.4%)
	R2M0 P0	0	51	1	10	0	17	0	5	1	1	0	0	0	0	86 (4.4%)
	R2M0 P1	3	1	3	0	2	0	1	0	1	0	0	0	0	0	11 (0.6%)
	R2M1 P0	1	26	0	66	0	4	1	34	1	2	1	3	0	0	139 (7.1%)
	R2M1 P1	0	0	3	1	5	0	1	1	2	0	0	0	0	0	13 (0.7%)
	R2MU P1^a^	0	0	0	1	0	0	0	0	0	0	0	0	0	0	1 (0.1%)
	R3M0 P0	0	4	0	2	0	2	0	4	0	1	0	2	0	0	15 (0.8%)
	R3M0 P1	2	0	5	0	0	0	3	0	0	0	5	0	2	0	17 (0.9%)
	R3M1 P0	0	1	0	4	0	4	0	6	0	0	0	6	0	0	21 (1.1%)
	R3M1 P1	1	0	5	0	2	1	0	1	0	0	3	2	5	0	20 (1.0%)
	R3MU P1^a^	0	0	1	0	0	0	0	0	0	0	0	0	0	2	3 (0.2%)
Total (%)		886 45.0%	591 30.0%	28 1.4%	322 16.4%	12 0.6%	29 1.5%	6 0.3%	53 2.7%	5 0.3%	4 0.2%	11 0.6%	13 0.7%	7 0.4%	2 0.1%	1,969 100.0%

^a^Unassessable level of maculopathy.

Images graded as those with any diabetic retinopathy included 61.6% of images taken with Eidon and 55.0% taken with 2-field mydriatic digital photography.

Using staged mydriasis, the sensitivity and specificity for any retinopathy were 97.5% with 95% CI (96.4–98.4%) and 82.3% with 95% CI (79.6–84.7%) respectively. There was near perfect inter-grader agreement in detecting any DR between graders assessing Eidon images (Cohen’s Kappa = 0.857).

Positive predictive value for any retinopathy was 87.1% with 95% CI (85.0–88.9%) and negative predictive value 96.4% with 95% CI (94.8–97.6%).

Images graded as those with referable diabetic retinopathy included 33.0% of images taken with Eidon and 23.6% taken with 2-field mydriatic digital photography.

Using staged mydriasis, the sensitivity and specificity for referable retinopathy were 92.7% with 95% CI (89.9–94.9%) and 85.4% with 95% CI (83.6–87.2%) respectively. There was substantial inter-grader agreement in detecting referable DR between graders assessing Eidon images (Cohen’s Kappa = 0.809).

Positive predictive value was 66.3% with 95% CI (62.5–69.9%) and negative predictive value 97.4% with 95% CI (96.4–98.2%).

If one counted the images that were unassessable on the Eidon but were gradable on the 2-field digital imaging as test positive the sensitivity and specificity for any DR was 97.5% (95% CI: 96.4–98.4%) and 80.6% (95% CI: 77.9–83.2%), and the sensitivity and specificity for referable DR was 92.7% (95% CI: 90.0–94.4%) and 84.2% (95% CI: 82.2–86.0%), respectively.

There were 16 eyes (0.8%) that were considered gradable on the Eidon but were unassessable on the reference standard 2-field digital images.

12 eyes (35.3%) that were unassessable using the Eidon camera had a pupil size of under 3 mm, which was felt to be the main reason for unassessabilty. The remaining 22 eyes (64.7%) were mostly unassessable due to media opacity (cataract, asteroid hyalosis and corneal scarring) but the exact reasons were not recorded on individual participants. A small number of younger patients had glare/reflection artefacts obscuring the macula and hence the images were unassessable.

The microaneurysm (MA) count was defined as non-inferior, when the number of microaneurysms detected within one disc diameter (1DD) of the centre of the fovea on images photographed using Eidon was equal to or higher than on the images taken using two 45-degree field digital cameras.

For staged-mydriasis images taken with Eidon, in 93.9% of cases 95% CI (92.8–94.9%) the Eidon was non-inferior in the detection of MA counts within 1DD of the centre of the fovea to the mydriatic digital photography used as a diabetic screening standard.

Out of 2029 images taken following staged-mydriatic protocol using the Eidon, the median number of MA counts was 0.0 with IQR (0.0–3.0) and mean MA count was 1.5 with SD (2.0).

The median number of MA counts for mydriatic digital photography equalled 0.0 with IQR (0.0–0.1) and mean MA count was 0.9 with SD (1.6).

To analyse the lesions detected outside the standard fields, the comparison has been made between images taken using staged dilation Eidon with unmodified 60-degree angle and staged-mydriasis Eidon with 2 ×45-degree overlay.

When the 60-degree angle was used, the additional diabetic retinopathy lesions Table 4  have been detected for 18 (0.9%) of eyes. The lesions were:

- 12 (0.6% of eyes) additional cases of non-referable diabetic retinopathy.
- 2 (0.1% of eyes) additional cases of referable diabetic retinopathy.
- 4 (0.2% of eyes) additional cases of higher level of referable retinopathy.

**Table 4:** Additional lesions detected outside the area of the two 45-degree fields.

		Staged mydriasis Eidon with 2 × 45° overlay																	
		No DR		Non referable DR		Referable DR												Unassessable	Total (%)
Staged mydriasis Eidon – 60° field		R0M0 P0	R0M0P1	R1M0P0	R1M0P1	R1M1P0	R1M1P1	R2M0P0	R2M0P1	R2M1P0	R2M1P1	R2MUP0*	R3M0P0	R3M0P1	R3M1P0	R3M1P1	R3MU P1*	Unassessable	
No DR	R0M0 P0	732	0	0	0	0	0	0	0	0	0	0	0	0	0	0	0	0	732 (36.1%)
	R0M0 P1	0	3	0	0	0	0	0	0	0	0	0	0	0	0	0	0	0	3 (0.2%)
Non-Referable DR	R1M0 P0	12	0	541	0	0	0	0	0	0	0	0	0	0	0	0	0	0	553 (27.3%)
	R1M0 P1	0	0	0	14	0	0	0	0	0	0	0	0	0	0	0	0	0	14 (0.7%)
Referable DR	R1M1 P0	0	0	0	0	315	0	0	0	0	0	0	0	0	0	0	0	0	315 (15.5%)
	R1M1 P1	0	0	0	0	0	7	0	0	0	0	0	0	0	0	0	0	0	7 (0.3%)
	R2M0 P0	0	0	1	0	0	0	84	0	0	0	0	0	0	0	0	0	0	85 (4.2%)
	R2M0 P1	0	0	0	0	0	0	0	8	0	0	0	0	0	0	0	0	0	8 (0.4%)
	R2M1 P0	0	0	0	0	1	0	0	0	137	0	0	0	0	0	0	0	0	138 (6.8%)
	R2M1 P1	0	0	0	0	0	0	0	0	0	9	0	0	0	0	0	0	0	9 (0.4%)
	R2MU P1^a^	0	0	0	0	0	0	0	0	0	0	1	0	0	0	0	0	0	1 (0.1%)
	R3M0 P0	0	0	0	0	0	0	1	0	0	0	0	15	0	0	0	0	0	16 (0.8%)
	R3M0 P1	0	0	0	1	0	0	0	0	0	0	0	0	17	0	0	0	0	18 (0.9%)
	R3M1 P0	0	0	0	0	0	0	0	0	1	0	0	0	0	21	0	0	0	22 (1.1%)
	R3M1 P1	0	0	0	0	0	0	0	0	0	1	0	0	0	0	20	0	0	21 (1.0%)
	R3MU P1^a^	0	0	0	0	0	0	0	0	0	0	0	0	0	0	0	2	0	2 (01%)
Unassessable	Unassessable	0	0	0	0	0	0	0	0	0	0	0	0	0	0	0	0	85	85 (4.2%)
Total (%)		744 36.7%	3 0.2%	542 26.7%	15 0.7%	316 15.6%	7 0.3%	85 4.2%	8 0.4%	138 6.8%	10 0.5%	1 0.1%	15 0.7%	17 0.8%	21 1.0%	20 1.0%	2 0.1%	85 4.2%	2,029 100.0%

^a^Unassessable level of maculopathy.

## Discussion

The Eidon camera, with 60-degree field, has performed accurately in detecting any level of diabetic retinopathy in the non-mydriatic mode with a sensitivity of 97.5% (95% CI 96.4–98.4%) and specificity of 82.3% (95% CI 79.6–84.7%) compared to mydriatic digital cameras that are being used in the English NHS DESP.

For images taken with staged mydriasis with the Eidon camera, in 93.9% (95% CI: 92.8–94.9%) of cases SCO was non-inferior in a detection of MA counts within 1DD of the centre of the fovea to the mydriatic digital photography used as a diabetic screening standard.

When detecting referable level of diabetic retinopathy, the sensitivity has decreased to 92.7% (95% CI 89.9–94.9%) but specificity increased to 85.4% (95% CI 83.6–87.2%).

There are clearly advantages in the detection of lesions outside the standard two 45-degree fields captured by the English NHS Diabetic Eye Screening Programme. We will be further analysing these results but preliminary analysis from Table 4 show that the Eidon camera detected an additional 0.6% of eyes with non-referable DR, 0.1% of eyes with referable diabetic retinopathy and 0.2% of eyes with a higher level of referable retinopathy.

There is no evidence of statistically significant difference (*p*-value = 1.000), between the proportion of unassessable eyes when using staged mydriasis with the Eidon and dilation with 2-field mydriatic digital reference standard devices.

The unassessable image rate without mydriasis of 4.2% (95% CI 1.2–2.3%) and the unassessable image rate using staged-mydriasis of 1.7% (95% CI 1.2–2.3%) in this predominantly White Caucasian population compares very favourably with previous reports of unassessable image rates for non-mydriatic digital cameras. For example, the original research on 45 degree non-mydriatic digital imaging cameras suggested [8, 9] that 19.7% and 26% might need mydriasis but, with the increasing age of the population, this was reported [10] by the Scottish Programme as having risen to 30% in a poster presented at the European Association for the Study of Diabetic Eye Complications in 2020. This study, with a similar age profile, reported that only 4.2% required dilation. This study only included 5% of people from Asian or Afro-Caribbean ethnic backgrounds, which is representative of the population of Gloucestershire. In 1985, Klein et al. [11] suggested that pupil colour may influence unassessable image rates and he recorded the pupil colour in his study as blue, hazel green or brown but the numbers in his study were insufficient to show any statistical significance. A study [12] by Gupta et al. in 2014 reported results of non-mydriatic photography in 500 patients with diabetes attending an endocrinology clinic in India. Two observers reported unassessable image rates of 30.6% and 31% in patients with a mean age of 52.97 ± 13.46 years.

With respect to the Afro-Caribbean population, an abstract [13] from the Association for Research in Vision and Ophthalmology (ARVO) conference in 2019 reported that it was not possible to obtain assessable images using non-mydriatic fundus photography in a substantial proportion of older, mostly African American individuals screened in the community.

In 2016, Silva reported [14] results from a screening programme for DR of American Indian and Alaska Native communities at 97 sites across 25 states. 35,052 eyes were imaged using non-mydriatic fundus photography (NMFP) and 16,218 eyes using the Optos Scanning Confocal Ophthalmoscope camera. Although the two groups of patients were different, the unassessable rate per patient was significantly lower with Optos imaging compared with NMFP (DR, 2.8% vs. 26.9% [*p* < 0.001].

The results of our study make the case for the use of this type of white light scanning confocal ophthalmoscope camera using staged mydriasis in Diabetic Eye Screening Programmes in the UK, but further work will be needed to determine the effectiveness of this approach in people of Asian and Afro-Caribbean ethnic backgrounds.

Further work is also needed to determine the cost-effectiveness of the use of this device in diabetic eye screening programmes.

## Summary

### What was known before

- Very little information was known about the use of a white light scanning confocal ophthalmoscope without eye drops in diabetic eye screening.

### What this study adds

- This study demonstrates a high performance of a white light scanning confocal ophthalmoscope without eye drops in diabetic eye screening.

## Supplementary information

Supplementary Table 1

Supplementary Table 2

Supplementary Table 3

## Data Availability

All data supporting the findings of this study are available within the paper and its Supplementary Information.

## References

[1] Sarao V, Veritti D, Borrelli E, Sadda SVR, Poletti E, Lanzetta P. A comparison between a white LED confocal imaging system and a conventional flash fundus camera using chromaticity analysis. BMC Ophthalmol. 2019;19:231. 31744471 10.1186/s12886-019-1241-8 PMC6862837

[2] Olvera-Barrios A, Heeren TF, Balaskas K, Chambers R, Bolter L, Tufail A, et al. Comparison of true-colour wide-field confocal scanner imaging with standard fundus photography for diabetic retinopathy screening. Br J Ophthalmol. 2020;104:1579–84. 32139499 10.1136/bjophthalmol-2019-315269

[3] Ashraf M, Hock KM, Cavallerano JD, Wang FL, Silva PS. Comparison of Widefield Laser Ophthalmoscopy and ETDRS Retinal Area for Diabetic Retinopathy. Ophthalmol Sci. 2022;2:100190. 36531579 10.1016/j.xops.2022.100190 PMC9754965

[4] ETDRS. Fundus photographic risk factors for progression of diabetic retinopathy. ETDRS report number 12. Early Treatment Diabetic Retinopathy Study Research Group. Ophthalmology. 1991;98:823–33. 2062515

[5] Scanlon PH, Aldington SJ, Leal J, Luengo-Fernandez R, Oke J, Sivaprasad S, et al. Development of a cost-effectiveness model for optimisation of the screening interval in diabetic retinopathy screening. Health Technol Assess. 2015;19:1–116. 26384314 10.3310/hta19740 PMC4780979

[6] Sivaprasad S, Vasconcelos JC, Prevost AT, Holmes H, Hykin P, George S, et al. Clinical efficacy and safety of a light mask for prevention of dark adaptation in treating and preventing progression of early diabetic macular oedema at 24 months (CLEOPATRA): a multicentre, phase 3, randomised controlled trial. Lancet Diabetes Endocrinol. 2018;6:382–91. 29519744 10.1016/S2213-8587(18)30036-6 PMC5908782

[7] Hajian-Tilaki K. Sample size estimation in diagnostic test studies of biomedical informatics. J Biomed Inf. 2014;48:193–204. 10.1016/j.jbi.2014.02.013 24582925

[8] Scanlon PH, Malhotra R, Thomas G, Foy C, Kirkpatrick JN, Lewis-Barned N, et al. The effectiveness of screening for diabetic retinopathy by digital imaging photography and technician ophthalmoscopy. Diabet Med J Br Diabet Assoc. 2003;20:467–74. 10.1046/j.1464-5491.2003.00954.x 12786681

[9] Ellingford MurgatroydH, Cox A, Binnie A, Ellis M, MacEwen JD, Leese CJ. GP. Effect of mydriasis and different field strategies on digital image screening of diabetic eye disease. Br J Ophthalmol. 2004;88:920–4. 15205238 10.1136/bjo.2003.026385 PMC1772219

[10] Styles C, Lee N, Black M, Ah-See K. Use of Dilating Drops in the Scottish Diabetic Retinopathy Screening Programme. European Association for the Study of Diabetic Eye Complications (EASDec). Eur J Ophthalmol. 2020;30:7–8.

[11] Klein R, Klein BE, Neider MW, Hubbard LD, Meuer SM, Brothers RJ. Diabetic retinopathy as detected using ophthalmoscopy, a nonmydriatic camera and a standard fundus camera. Ophthalmology. 1985;92:485–91. 4000642 10.1016/s0161-6420(85)34003-4

[12] Gupta V, Bansal R, Gupta A, Bhansali A. Sensitivity and specificity of nonmydriatic digital imaging in screening diabetic retinopathy in Indian eyes. Indian J Ophthalmol. 2014;62:851–6. 25230960 10.4103/0301-4738.141039 PMC4185162

[13] Gajwani P, Zhao D, Guallar E, Wahl M, David J, Dosto N, et al. Ungradable non mydriatic fundus photography in community eye screening. Investigative Ophthalmol Vis Sci. 2019;60:5569.

[14] Silva PS, Horton MB, Clary D, Lewis DG, Sun JK, Cavallerano JD, et al. Identification of Diabetic Retinopathy and Ungradable Image Rate with Ultrawide Field Imaging in a National Teleophthalmology Program. Ophthalmology. 2016;123:1360–7. 26949120 10.1016/j.ophtha.2016.01.043

